# Intermittent blood flow restriction with low-load resistance training for older adults with knee osteoarthritis: a randomized, controlled, non-inferiority trial protocol

**DOI:** 10.1186/s13063-024-08203-9

**Published:** 2024-05-31

**Authors:** Qiao-Mei Hong, Hao-Nan Wang, Xi-Hui Liu, Wen-Qi Zhou, Xiao-Bing Luo

**Affiliations:** 1 Department of Sport Medicine, Sichuan Province Orthopedic Hospital, Chengdu, Sichuan Province China; 2 Sports Medicine Center, West China Hospital, Sichuan University, Chengdu, China; 3 Department of Orthopedics and Orthopedic Research Institute, West China Hospital, Sichuan University, Chengdu, China

**Keywords:** Knee osteoarthritis, Blood flow restriction, Resistance training, Vascular occlusion, Rehabilitation

## Abstract

**Background:**

Knee osteoarthritis (KOA) is a chronic musculoskeletal disorder characterized by pain and functional impairment. Blood flow restriction (BFR) with low-load resistance training (LLRT) demonstrates a similar improvement in clinical outcomes to high-load resistance training (HLRT) in treating KOA. It has not been established whether intermittent blood flow restriction (iBFR) with LLRT can lead to clinical outcomes that are comparable to those produced by continuous blood flow restriction (cBFR) with LLRT and HLRT. The aim of the proposed study is to evaluate the efficacy of iBFR with LLRT on pain, Western Ontario and McMaster Universities Osteoarthritis Index (WOMAC), muscle strength, muscle mass, physical function, perceptions of discomfort and effort, and adherence in KOA patients.

**Methods:**

This is a three-arm, non-inferiority, randomized controlled trial utilizing blinded assessors. Two hundred thirteen participants will be randomly allocated to one of the following three groups: iBFR group—receiving 4 months of LLRT with iBFR, twice weekly (*n* = 71); cBFR group—receiving 4 months of LLRT with cBFR, twice weekly (*n* = 71); or HLRT group—receiving 4 months of HLRT without BFR, twice weekly (*n* = 71). The primary outcome is pain. The secondary outcomes include the WOMAC, muscle strength, muscle mass, physical function, perceptions of discomfort and effort, and adherence. Pain and WOMAC will be measured at the baseline and 4 and 12 months after randomizations. Muscle strength, muscle mass, and physical function will be measured at the baseline and 4 months after randomizations. The perceptions of discomfort and effort will be measured during the first and final sessions.

**Discussion:**

BFR with LLRT has a similar improvement in clinical outcomes as HLRT. However, cBFR may cause elevated ratings of perceived exertion and local discomfort, compromising patient tolerability and treatment adherence. If iBFR with LLRT could produce improvement in clinical outcomes analogous to those of HLRT and iBFR with LLRT, it could be considered an alternative approach for treating patients with KOA.

**Trial registration:**

Chinese Clinical Trial Registry ChiCTR2300072820. Registered on June 26, 2023.

**Supplementary Information:**

The online version contains supplementary material available at 10.1186/s13063-024-08203-9.

## Background

Knee osteoarthritis (KOA) is a chronic musculoskeletal disorder characterized by pain, stiffness, and functional disability [1]. The global prevalence of KOA is high and expected to continue rising substantially, leading to a significant economic burden on healthcare systems [2]. Clinical guidelines of KOA guidelines consistently recommend exercise-based programs as the first-line intervention, based on clinical evidence of their efficacy [3, 4]. Previous studies have indicated that muscle weakness is strongly associated with symptoms and physical function in KOA [5–7]. Additionally, muscle mass in the lower limbs influences the severity of symptoms in patients with KOA [8]. Resistance training (RT) is a core component of exercise programs for KOA and is intended to increase muscle strength and promote muscle hypertrophy [9]. According to the American College of Sports Medicine’s recommendations, a minimum resistance load of 70% and 60% of an individual’s one-repetition maximum (1RM) is necessary to promote muscle hypertrophy and gain muscular strength, respectively [10]. However, high-load resistance training (HLRT) has the potential to worsen joint damage, particularly in cases where there is pre-existing deterioration, and may be less well-tolerated due to knee pain in patients with KOA [11].

Blood flow restriction (BFR) with low-load resistance training (LLRT) at 20–30% of 1RM enhances muscle strength and mass gains while minimizing harmful joint loading. BFR induces local ischemia and hypoxia by applying a pressurized cuff around the proximal limb region, partially obstructing arterial blood flow and restricting venous return [12]. Although the mechanisms underlying the chronic adaptations to BFR training remain inconclusive, it has been proposed that the augmentation arises from elevated physiological metabolic stress [13, 14] and increased recruitment of type II muscle fibers [15]. In older populations, decreases in lower extremity function are significantly correlated with decreases in muscle strength [16]. BFR with LLRT effectively induces muscle hypertrophy comparable to that achieved with HLRT [17]. A previous meta-analysis has indicated that BFR training is a relatively safe approach, producing comparable advantages in pain relief, functional improvement, and muscle strength gains as HLRT [18]. BFR with LLRT produces better improvements in muscle strength than LLRT alone in patients at risk for KOA [19, 20], while its efficacy in increasing muscle strength, muscle mass, and function in patients with KOA is similar to that of HLRT [21, 22].

Despite substantial research affirming the efficacy of BFR training, only one review has focused on optimizing BFR training variables, such as cuff pressure, exercise type, duration, and intensity [23]. Although studies have begun to explore the impact of these parameters (e.g., cuff pressure) on BFR interventions for KOA [24], at present, we still have no clue as to the optimal BFR approach. Typically, the BFR cuff is inflated prior to commencing BFR training and remains inflated during the sessions, which is referred to as continuous blood flow restriction (cBFR). This cBFR approach may be accompanied by elevated ratings of perceived exertion and local discomfort, resulting in poor tolerability and adherence [25–27]. Therefore, it has been proposed that intermittent cuff pressure deflation during rest periods between sets, known as intermittent blood flow restriction (iBFR), may lead to decreased pain and perceived exertion during BFR training. Most previous studies of BFR training in patients with KOA have used cBFR, but it has been reported that this approach causes knee pain or discomfort [19, 20, 22, 28]. The tolerability of iBFR training makes this approach more suitable for older adults, particularly individuals with knee pain due to KOA.

To date, the efficacy of iBFR in stimulating muscle adaptation to enhance clinical outcomes in KOA patients remains undetermined. A previous study showed that compared to iBFR training, cBFR training leads to greater metabolic stress accumulation, manifested as increased inorganic phosphate and decreased intramuscular pH levels after multiple training sets [29]. This effect can be attributed to the intermittent release of cuff pressure in iBFR training, alleviating the accumulation of metabolites during iBFR training. However, it has been recently postulated that in BFR training, the anabolic effects of metabolites may arise solely from increased motor unit recruitment [30]. Consequently, excessive metabolic stress could be redundant if it surpasses the recruitment threshold for high-threshold motor units. In fact, no discernible difference in muscular activity was observed between cBFR and iBFR with LLRT during BFR training sessions [31]. During chronic training periods, iBFR training yielded benefits comparable to cBFR training in both thigh muscle cross-sectional area and lower body lean mass [32]. Furthermore, iBFR training demonstrated similar efficacy in enhancing isometric and isokinetic muscle strength compared to cBFR training [32]. Conversely, elevated metabolic stress could activate group III/IV afferents, potentially causing pain during BFR training sessions [33, 34]. This could explain why cBFR training evoked greater discomfort compared to iBFR training [35].

Although results from both acute and chronic studies show that iBFR is a suitable alternative to cBFR for training in a healthy population [29, 32], the effects have not yet been corroborated in patients with KOA. Determining the differential clinical intervention effects of iBFR and cBFR in the KOA population could help professionals optimize BFR treatment for KOA and develop future guidelines for BFR in musculoskeletal disorders. This may help to improve the efficiency of conservative treatment for patients with KOA, improve patients’ quality of life, and further reduce healthcare costs. The purpose of the current study is to investigate the efficacy of iBFR with LLRT on pain, Western Ontario and McMaster Universities Osteoarthritis Index (WOMAC), muscle mass, muscle strength, and physical functions while monitoring discomfort, effort, and adverse events compared to cBFR with LLRT and HLRT in KOA patients. We hypothesize that there will be no difference among the cBFR, iBFR, and HLRT protocols in pain, WOMAC, muscle mass, muscle strength, and physical function. Our secondary hypothesis is that the discomfort and effort in the cBFR and HLRT groups will be greater than in the iBFR group during the first and final training sessions.

## Methods

### Trial design and setting

**Fig. 1:**
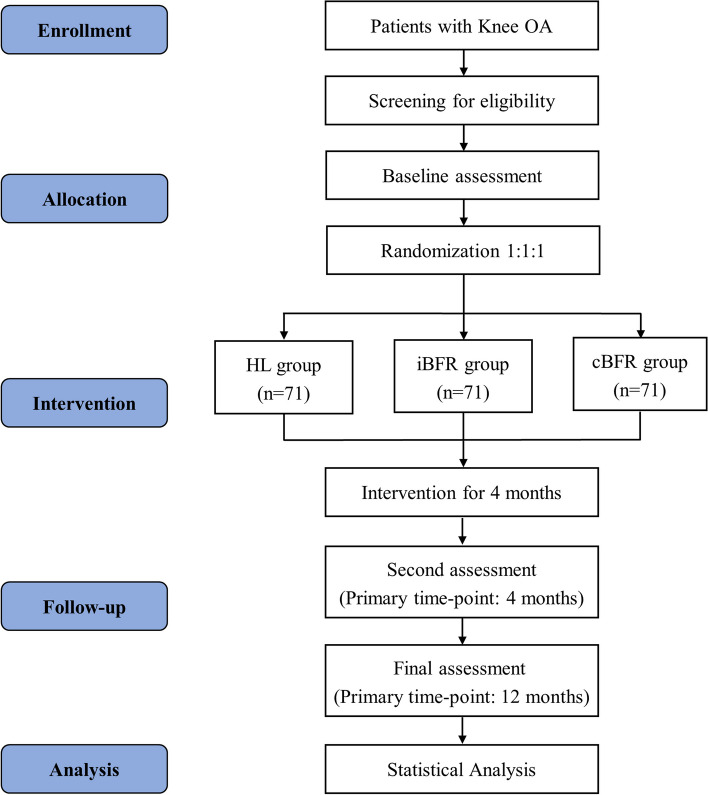
Flow diagram of the planned study. KOA, knee osteoarthritis; HL, high load; iBFR, intermittent blood flow restriction; cBFR, continuous blood flow restriction

This study is a randomized controlled trial with three parallel groups: This study adopts a randomized controlled trial design featuring three parallel groups: iBFR, cBFR, and HL (Fig. 1). Participant recruitment will be through online advertisements, community outreach, and outpatient referrals.

The study protocol received approval from the Ethics Committee of Sichuan Province Orthopedic Hospital (Approval No. KY2022-028–01) and is registered with the Chinese Clinical Trial Registry (ChiCTR2300072820). Measurements will be taken at three time points: baseline, 4 months post-intervention, and 12 months post-intervention. We chose a 4-month intervention period because it provides sufficient time to observe significant changes in OA-related pain and function [36].

**Fig. 2:**
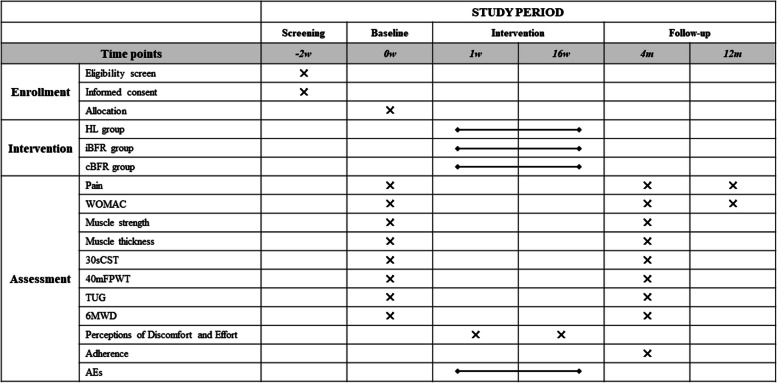
Schedule of enrollment, intervention, and assessment (SPIRIT figure). HL, high load; iBFR, intermittent blood flow restriction; cBFR, continuous blood flow restriction; WOMAC, Western Ontario and McMaster Universities Osteoarthritis Index; 30sCST, 30-s chair-stand test; 40mFPWT, 40-m fast-paced walk test; TUG, timed up and go test; 6MWD, 6-min walk distance test; AEs, adverse events

The protocol of our study is developed and reported following the Standard Protocol Items: Recommendations for Interventional Trials (SPIRIT) statement [37] (Additional file 1 and Fig. 2) and TIDieR guideline (Additional file 2). The results of the current study will be reported in accordance with the CONsolidated Standards of Reporting Trials (CONSORT) guidelines [38].

Participants will be recruited at the Sichuan Province Orthopedic Hospital (SPOH), and all data collection will be conducted at SPOH. SPOH is a prominent non-profit institution in Sichuan, serving as the approved hospital for national team athletes by the Chinese Olympic Committee. SPOH’s Sport Medicine Department boasts one of China’s premier therapeutic exercise centers, specializing in both primary and specialty care for national athletes. Annually, the hospital attends to over 700,000 outpatient and emergency cases, with seasoned clinicians experienced in treating degenerative bone and joint conditions.

### Participants

The inclusion criteria are as follows: (1) 50–75 years of age, (2) unilateral or bilateral KOA diagnosed according to the American College of Rheumatology clinical classification system [10, 39], (3) radiologic evidence of KOA demonstrating Kellgren-Lawrence grade II or III [40], (4) average overall knee pain severity of 40 or greater on a 100-mm visual analog scale (VAS), and (5) adequate Mandarin language skills to complete the Chinese version of WOMAC and the written informed consent.

The exclusion criteria are as follows: (1) a history of knee surgery or scheduled surgery; (2) a history of any invasive procedure in the affected knee, including arthroscopy or intra-articular injection in the past 12 months; (3) a history of physiotherapy or a strengthening exercise program of the affected knee in the past 6 months; (4) use of oral nonsteroidal anti-inflammatory drugs in the past 3 months; (5) any neurological, heart, or vascular disease, such as blood coagulation disorders; or (6) other acute or chronic conditions that will affect physical or cognitive functions.

### Enrollment, randomization, and blinding

All potential participants will initially be screened for the eligibility criteria via telephone. Individuals who pass the initial screening and are interested in the study will undergo a secondary screening in person. Subsequently, all participants will sign a written informed consent and complete the baseline assessment. Following the baseline assessment, participants will be randomly allocated to iBFR, cBFR, and HL groups in a 1:1:1 ratio, separately. The block randomization method was used, with each block containing nine allocations. An independent researcher will create the allocation schedule using a computerized random number generator (www.randomizer.org). The research assistants responsible for enrollment were not informed about the assessment and intervention. The outcome assessors will be blinded in the current study. Blinding will be revealed only in the event of a serious adverse event occurs.

### Intervention procedure

All participants in the intervention groups will complete 32 exercise sessions, conducted twice weekly over 4 months. Participants from all groups will continue their regular OA medical care throughout the study duration. For ethical reasons, participants will be allowed to use acetaminophen and topical diclofenac sodium gel up to a limit of 3 g daily as rescue medication. Usage must be documented and discontinued at least 72 h prior to assessments of clinical outcomes.

#### Determination of one repetition maximum test

All participants will perform the 1RM to determine the resistance load on the leg press and leg extension machine (Precor, Precor USA Inc., Woodinville, WA, USA). Instead of the direct 1RM test, the participants will perform the 7–10 RM test, assessing the maximum load one can lift within 7–10 repetitions in a single set. This method is chosen due to the participants being elderly with joint disorders. This load will then be used to estimate the 1 RM using the Bryzcki formula [41]: estimated 1RM = weight/(1.0278 − 0.0278 × reps). The 7–10 RM test is able to accurately estimate the 1 RM for leg press [42] and knee extension exercises [43]. The exercise resistance will be adjusted every 4 weeks by re-assessing the participant’s 1RM to minimize the impact of physiological adaptation on individual 1RM.

#### Determination of LOP

The limb occlusion pressure (LOP) will be assessed to determine the individualized pressure of the BFR cuff by using the portable color Doppler ultrasound (CX50, Philips Healthcare, Best, Netherlands) during the baseline assessment and re-evaluated bi-weekly. In the assessment, participants will be placed in relaxed and supine positions. A pneumatic cuff (7 cm width and 56 cm length) will be affixed to the proximal thigh, and the Doppler ultrasound probe will be positioned at the dorsal ankle to monitor the pedal pulse. The cuff is first inflated to match the participant’s systolic brachial blood pressure for around 10 s. It is then progressively inflated in 10-mmHg intervals until the elimination of the pedal pulse is detected by the Doppler ultrasound. Once the pedal pulse ceases, the cuff will be gradually deflated until the pulse returns. The pressure at which the pedal pulse is entirely interrupted is recorded as the LOP.

#### BFR protocols

In this study, the iBFR group will perform at 70% LOP during the resistance training as recommended by previous study [22, 44]. The cuffs will be inflated immediately prior to the first set of leg press and seated knee extension exercises for both iBFR and cBFR groups. In the cBFR group, the cuff stays inflated throughout the exercise, including the rest periods between sets. In the iBFR group, the cuff is deflated during each set’s rest period and is quickly reinflated to the target pressure 20–30 s into the rest, ensuring the entire rest period does not exceed 45 s. The cuffs are deflated between exercises for both the iBFR and cBFR groups.

#### Exercise protocols

Each training session will be conducted under the one-on-one guidance of a well-trained physiotherapist. Individual time slots will be allocated for each participant. Each session comprises a 10-min warm-up with static cycling, a 30-min exercise program, and a 15-min cooldown. The exercise program includes eight exercises: leg extension, leg press, hip abduction, calf exercise, seated calf raises, sensori-motor training, and core exercises (Table 1).

**Table 1:** Treatment protocol performed by the HL group and the BFR groups

HL group	iBFR group	cBFR group
Hamstrings stretching, 3 repetitions of 30 s	Hamstrings stretching, 3 repetitions of 30 s	Hamstrings stretching, 3 repetitions of 30 s
Bridge with isometric contraction of the transversus abdominis-CORE training, 3 repetitions of 30 s^c^	Bridge with isometric contraction of the transversus abdominis-CORE training, 3 repetitions of 30 s^c^	Bridge with isometric contraction of the transversus abdominis-CORE training, 3 repetitions of 30 s^c^
Hip abduction with weights (side lying), 3 sets of 10 repetitions^c^	Hip abduction with weights (side lying), 3 sets of 10 repetitions^c^	Hip abduction with weights (side lying), 3 sets of 10 repetitions^c^
Calm exercises (side lying) with an elastic band, 3 sets of 10 repetitions^c^	Calm exercises (side lying) with an elastic band, 3 sets of 10 repetitions^c^	Calm exercises (side lying) with an elastic band, 3 sets of 10 repetitions^c^
Calf raises with weights (standing), 3 sets of 10 repetitions^c^	Calf raises with weights (standing), 3 sets of 10 repetitions^c^	Calf raises with weights (standing), 3 sets of 10 repetitions^c^
Sensori-motor training (standing) at mini-trampoline, 3 repetitions of 30 s	Sensori-motor training (standing) at mini-trampoline, 3 repetitions of 30 s	Sensori-motor training (standing) at mini-trampoline, 3 repetitions of 30 s
Leg press (machine), 0–60°, 1 set of 30 repetitions, and 3 sets of 15 repetitions^a^	Leg press (machine), 0–60°, 4 sets of 10 repetitions^a,^^d^	Leg press (machine), 0–60°, 4 sets of 10 repetitions^a,e^
Seated knee extension (machine), 90–0° of knee flexion, 1 set of 30 repetitions, and 3 sets of 15 repetitions^a^	Seated knee extension (machine), 90–0° of knee flexion, 4 sets of 10 repetitions^b,^^d^	Seated knee extension (machine), 90–0° of knee flexion, 4 sets of 10 repetitions^b,e^

^a^Load is 30% of the 1-repetition maximum

^b^Load is 60% of the 1-repetition maximum

^c^The load will be adjusted every 4 weeks to maintain an effort of perception between 6 and 7 on the Borg scale

^d^Intermittent blood flow restriction will be conducted

^e^Continuous blood flow restriction will be conducted

The leg extension and leg press exercises will be performed on individual legs to prevent unequal loading. To reduce patellofemoral joint stress, these two exercises have a limited range of motion. The leg press will be performed between 0 and 60° of knee flexion, and the leg extension will be performed between 90 and 45° of knee flexion [45]. The exercise protocol for the leg press and leg extension in the HL group involves 4 sets of 10 repetitions at 60% 1RM, with 45-s intervals between sets [19, 21, 46]. In both iBFR and cBFR groups, the loads for the leg press and knee extension exercises are set at 30% of the 1RM. Participants will complete 4 sets: one set of 30 repetitions (or until exhaustion) and 3 sets of 15 repetitions with a 45-s interval between sets [20]. The modified 10-point Borg scale will be adopted to gauge perceived effort during hip abduction, calf exercises, seated calf raise, and core exercises. To maintain a perceived effort between 6 and 7 on the Borg scale, the intensity of exercises will be modulated using weights, elastic bands, or duration adjustments. Participants are encouraged to continue with their BFR training or RT after the intervention has finished, up until the final outcome measurements are collected at 12 months.

#### Pain monitoring

The pain intensity will be monitored by a VAS during the exercise protocols. If the pain intensity surpasses 20 mm/100 mm on the VAS, the load or intensity of exercise will be decreased by 20% [46]. Participants will also be asked to report whether their pain increased following the adjustment of the load or intensity. If participants report increased pain over three sessions, the load or intensity will be reverted to its previous level.

### Outcomes

#### Primary outcome measures

##### Pain

The level of rest pain and worst pain is evaluated using the VAS, which ranges from 0 to 100 mm. A score of “0 mm” indicates no pain, and a score of “10 mm” indicates the most severe pain that can be tolerated [47]. The VAS has undergone assessment for reliability, validity, and responsiveness in rating pain and is an effective pain assessment tool widely applied in patients with OA. The VAS has a test–retest reliability of 0.97 in patients with KOA [48]. This study will use VAS to evaluate rest and worst pain experienced at baseline, 4 months, and 12 months follow-up.

#### Secondary outcome measures

##### The Western Ontario and McMaster Universities Osteoarthritis Index

The Western Ontario and McMaster Universities Osteoarthritis Index (WOMAC) is a 24-item self-report questionnaire that evaluates joint pain, stiffness, and physical function related to KOA [49]. Each question presents 5 response options on a scale where 0 = none and 4 = extreme, and the lower the score represents better knee joint functions. The Chinese version of the WOMAC has demonstrated both validity and reliability, as well as sensitivity to changes in patients with KOA [50]. The WOMAC will be assessed at baseline, 4 months, and 12 months follow-up.

##### Muscle strength

The quadriceps muscle strength will be evaluated by strength test using an isokinetic test system (IsoMed 2000, D&R Ferstl GmbH, Hemau, Germany). Participants will be securely positioned on a dynamometric chair at a 90° angle. Rigid belts will limit the compensatory movements of the torso and thighs. The dynamometer’s axis will be aligned with the knee joint’s rotation center using a laser device. The range of motion will then be set individually for the participants by actively extending and flexing their knees to maximum ranges. Before the formal test, participants will perform three submaximal repetitions for familiarization. In the formal test, participants will execute five consistent flexion and extension motions using the concentric-concentric contraction model at three angular velocities: 60°/s, 90°/s, and 120°/s, without gravity compensation. The participants will be encouraged to perform at their maximum effort in the testing process. Data will capture the peak torque (in Newton meters), peak torque relative to body weight (in Newton meters/kg), and power (in watts). Previous research has shown that isokinetic knee muscle strength assessment has a test–retest reliability of 0.94 [51]. Muscle strength will be measured at baseline and 4 months follow-up.

##### Muscle thickness

Quadriceps muscle thickness will be evaluated using a portable Doppler ultrasound [46, 52]. The ultrasound probe will be positioned on the mid-belly of the vastus medialis, vastus lateralis, and rectus femoris, ensuring no skin depression. The images will be saved, and then the muscles will be measured by an independent assessor. The distance from the adipose tissue-muscle interface to the muscle-bone interface will be measured three times and averaged to determine the muscle thickness for each muscle. Quadriceps muscle thickness is the cumulative thickness of these three muscles. The correlations between the ultrasound and magnetic resonance imaging scans for muscle thickness of the vastus medialis, vastus lateralis, and rectus femoris are 0.86, 0.94, and 0.86, respectively [53, 54]. The muscle thickness will be measured at baseline and 4 months follow-up.

##### Physical function

The physical function tests include the 30-s chair-stand test (30sCST), 40-m fast-paced walk test (40mFPWT), timed up and go (TUG) test, and 6-min walk distance test (6MWD) [55]. In the 30sCST, participants stand and sit as many times as they can within 30 s, counting each full sit as one repetition [56]. The test–retest reliability of the 30-s chair stand test is 0.9 [57]. The 40mFPWT measures the time it takes for an individual to walk a distance of 40 m as quickly as possible without running. The 40mFPWT shows optimal levels of both the inter-rater and intra-rater reliability (0.96 and 0.92, respectively) in patients with osteoarthritis [58]. In the TUG test, participants rise independently from a 45-cm high armchair, walk straight for 3 m, turn around, walk back, and sit. The entire process’s duration is recorded [59]. TUG test has a good intra- and inter-rater reliability (0.97 and 0.96, respectively) for KOA patients graded I–III on the Kellgren-Lawrence scale [60]. For the 6MWD test, participants walk back and forth in a long, straight hallway for 6 min, aiming to cover maximum distance. The minimal clinically important difference (MCID) for the 6MWD ranges between 26 and 55 m [61]. Participants will be required to practice three times before the formal testing [62]. Each physical function test will be measured three times, and the average of three measurements will be analyzed. All physical function tests will be measured at baseline and 4 months follow-up.

##### Perceptions of discomfort and effort

Perceptions of both discomfort and exertion will be measured following each set of leg press and leg extension exercises during the first and final sessions (week 1 and week 16). Perceptions of pain will be recorded using 100-mm VAS, while perceived exertion will be recorded using a modified 10-point Borg scale. The subjects will be reminded that discomfort and effort are different sensations in exercise and instructed on how to distinguish the feeling of discomfort and effort during the familiarization at the first session [63]. Specifically, exertion is the general sensation of fatigue from the exercise, while pain is considered as unpleasant feeling within the local muscles. Participants will be asked to confirm their understanding of the differences between the ratings. The mean values from all sets of the leg press and leg extension exercises will be incorporated into the statistical analysis.

##### Exercise adherence

Exercise adherence will be reported descriptively as a percentage of the total number of prescribed intervention sessions completed [64].

##### Adverse events

During the consent process, patients will be informed of the potential adverse events (AEs) and instructed to notify a researcher when AEs occur. Participants will be queried about any AEs, and they will also be prompted to recall such events during the 4- and 12-month testing visits. By the end of the trial, all AEs will be recorded and documented, regardless of whether they are associated with the study or not. Potential AEs associated with BFR or resistance training include muscle soreness, subcutaneous hemorrhage, and numbness. Once AEs are reported, physiotherapists and relevant specialists will categorize them as treatment-related or not and assess their severity within 24 h. The investigator will promptly take appropriate actions in response to any adverse events that arise during the study, as dictated by the patient’s condition. All participants in the trial are covered by clinical trial insurance. Participants will have access to medical care provided by the study team or referred to appropriate medical professionals as needed.

### Sample size estimation

The sample size is determined by using PASS (V.21.0, NCSS, LLC, Kaysville, UT, USA) to detect non-inferiority in between-group differences for pain measured on the VAS scale. The non-inferiority test method is based on performing a one-sided two-sample *t*-test sample size calculation, multiplying the variance by a factor 1-*ρ*2, where *ρ* represents the Pearson correlation coefficient between the baseline and follow-up outcome measures [65]. For a change in VAS, a non-inferiority margin of 16 mm is chosen as this is less than the MCID of 17.5 mm on a 100-mm VAS for KOA patients [66]. The significance level is set at one-sided 0.0083 (Bonferroni correction for three pairwise comparisons). Assuming standard deviations (SD) of changes from baseline of 28 mm for VAS and correlations of *ρ* = 0.3 between baseline and follow-up [67, 68], 15% drop rate, the final sample size will be 71 patients for each group.

### Statistical analyses

The data will be analyzed by statisticians who are blinded to the process of assignment and interventions. The intention-to-treat (ITT) principle will be adopted in this trial. There may be missing data in the follow-up outcome measures due to dropout, a missed interim assessment, or patient non-response. Missing data will be multiply imputed using chained equations with predictive mean matching while imputing data for each group separately. Estimates based on 10 imputed data sets were then combined using Rubin rules [69]. If no statistically significant differences are found among groups and there’s a potential covariance in the baseline data, covariance analysis will be used for adjustments. For outcomes with repeated measurement, they will be compared at all following time point by using a linear mixed model with repeated measurement. The Tukey post hoc test will be used for multiple comparisons purposes. All statistical analyses will be performed using the SAS software (V 9.3, SAS Institute, Cary, NC, USA). The data will be presented as mean ± SD or median depending on whether the distribution is normal or not. The significance level for all analyses will be set at 0.05.

### Withdrawal criteria

All participants will be informed of their right to withdraw at any time. They will also be assured that no biological specimens will be collected for storage during the consent process. Participants may choose or be asked to leave the study under the following cases: (1) participants voluntarily choose to leave; (2) a severe adverse event occurs; (3) unforeseen events, like injuries, prevent the continuation of the intervention; and (4) participants receive other therapy. If a participant decides to withdraw, the researchers will record the reason and date of the withdrawal. Participants who discontinue the intervention will still be encouraged to attend follow-up assessments.

### Data management

Data will be meticulously documented using both printed forms and electronic case report forms (eCRFs). Participant data will be anonymized by removing any personally identifiable information from the dataset and replacing it with unique identifiers. Only outcome assessors will access the eCRFs, and an independent assessor will double-check all input data. Once data is input and verified in the eCRF, it becomes unmodifiable. Only statisticians will access the database to conduct the final statistical analyses. All study personnel, including researchers and assistants, will be required to sign confidentiality agreements.

### Quality control

A standard operation procedure manual will be developed to standardize the administration of the subjective questionnaires, objective performance tests, and AE reports throughout the trial. All researchers involved will undergo standardized training and assessments to minimize the measurement bias. An independent Data and Safety Monitoring Board, comprising experts in orthopedics, physiotherapy, methodology, and statistics, will oversee each study phase for quality assurance. Four weeks post the treatment of the first patient, the board will review the trial progress and determine if early study termination is warranted due to adverse events or data validity concerns. The Research Administration of Sichuan Province Orthopedic Hospital will be responsible for verifying the accuracy of the data collection. Every 2 weeks, the Sichuan Province Orthopedic Hospital’s Ethics Committee will check for any protocol deviations. Both online and onsite monitoring will be employed to uphold the trial’s quality by reviewing its processes. In case of any protocol changes, participants, the ethics committee, and the Chinese Clinical Trial Registry Center will be notified via email.

## Discussion

In recent years, BFR training has emerged as a promising therapeutic exercise for KOA because it stimulates muscle strength and muscle mass with low joint loading. However, there is currently no guideline providing recommendations for BFR training in the KOA population. In the present study, we will investigate the effectiveness of intermittent BFR with LLRT on the clinical outcomes including pain, WOMAC, muscle strength, muscle mass, physical function, and the perceptions of discomfort and effort during the BFR training in individuals with KOA. The findings of this study will provide valuable information about the suitability of iBFR with LLRT for the KOA population, which can be useful for physiotherapists in clinical practice.

Most previous studies investigating the effect of BFR in patients with KOA either used cBFR [22, 28] or did not specify the method [21]. In healthy individuals, it was found that iBFR and cBFR have a similar effect on muscle strength and muscle mass [32, 70, 71]. However, the participants subjected to iBFR experience less discomfort compared to those in cBFR during the BFR training intervention [72]. Patients with higher pain self-efficacy tend to exhibit greater pain tolerance [73], potentially boosting their adherence to BFR training. The HLRT has been the traditional exercise intervention for KOA patients [74]. For this reason, we will establish a control group (HLRT) to evaluate the difference between the iBFR with LLRT and HLRT, which will be beneficial to determine the feasibility of iBFR in clinical practice. Although a previous study showed that BFR with LLRT and HLRT were equally effective in improving muscle strength, quadriceps muscle mass, and functionality in KOA [22], the unmatched total exercise volume (load × repetitions × sets) between these two interventions may have hindered the true effectiveness. Therefore, we ensured comparable total exercise volume across all three groups.

This study was designed and reported in accordance with the SPIRIT statement, adhering to rigorous methodological standards. The randomization, allocation concealment, blinding of the assessor, and ITT approach will be applied in the present study. Due to the nature of BFR and exercise intervention, it was not possible to blind the participants and physiotherapists. All clinical outcomes will be evaluated at the end of the intervention to investigate the effect of iBFR with LLRT in the mid-term (3–6 months) follow-up. The objective outcomes, such as pain and WOMAC, will also be measured at 12 months from the baseline to assess the long-term effects of BFR with LLRT. In addition, we will assess the perceptions of discomfort and effort during the first and final session, which is beneficial to determine the suitability of iBFR throughout the exercise intervention.

The exercise intervention was designed and reported in accordance with the TIDieR guideline. The prescription of exercise follows the recommendation by the American College of Sports Medicine [75], including frequency, intensity, time, type, volume, pattern, and progression. Notably, the exercise load will be tailored based on the strength of each individual. To ensure participant safety and tolerance, a submaximal strength test will be conducted to estimate the 1RM, instead of a direct maximal strength test measurement. Currently, no specific guidelines exist for BFR training that dictate the precise parameters for its use. Therefore, the BFR parameters used in this study are based on recommendations and protocols from previous studies [23, 76], which include cuff width, cuff strapping location, individual LOP, and body position during LOP measurement. We will measure the LOP for participants and adjust the cuff pressure based on a percentage of the LOP. This ensures a consistent degree of BFR, as a higher degree of BFR induces a stronger metabolic response [44]. To maintain the correct position of the strapping cuff, it cannot be removed after deflation.

Several limitations exist in this study. It is not feasible to blind the participants and therapists involved in the exercise intervention; however, blinding of the assessor and statistician will be carried out for the outcome measures. In addition, assessments will only be conducted at the 4 and 12 months after randomization for pain and WOMAC, thus limiting the determination of long-term effects (≥ 12 months) of the iBFR with LLRT on muscle strength, muscle mass, and physical function. Due to the fact that this trial is a non-inferiority randomized controlled trial comparing the clinical efficacy of iBFR with LLRT versus HLRT and cBFR with LLRT, it does not include a control group for usual care. Finally, similar to past research, the LOP in this study was assessed in the resting position, which may not accurately reflect the occlusion pressure during physical activity due to hemodynamic variations.

## Trial status

The currently approved version of the protocol is version 1.0 dated October 2022. Recruitment is still in progress and will be completed by June 2024.

### Supplementary Information

Additional file 1: SPIRIT Checklist.

Additional file 2: TIDieR Checklist.

## Data Availability

The results and relevant data of this study will be published through academic conferences and scientific papers. The datasets can be obtained from the corresponding author upon reasonable request.

## Abbreviations

KOA: Knee osteoarthritis
LLRT: Low-load resistance training
HLRT: High-load resistance training
BFR: Blood flow restriction
iBFR: Intermittent blood flow restriction
cBFR: Continuous blood flow restriction
VAS: Visual analog scales
WOMAC: Western Ontario and McMaster Universities Osteoarthritis Index
1RM: One-repetition maximum
LOP: Limb occlusion pressure
30sCST: Thirty-second chair-stand test
40mFPWT: Forty-meter fast-paced walk test
TUG: Timed up and go test
6MWD: Six-minute walk distance test
MCID: Minimal clinically important difference
AEs: Adverse events
ITT: Intention-to-treat
SD: Standard deviation
eCRFs: Electronic case report forms
